# Blockade of dual immune checkpoint inhibitory signals with a CD47/PD-L1 bispecific antibody for cancer treatment

**DOI:** 10.7150/thno.79367

**Published:** 2023-01-01

**Authors:** Rongjuan Wang, Chang Zhang, Yuting Cao, Junchao Wang, Shasha Jiao, Jiao Zhang, Min Wang, Peipei Tang, Zijun Ouyang, Wenlu Liang, Yu Mao, An Wang, Gang Li, Jinchao Zhang, Mingzhu Wang, Shuang Wang, Xun Gui

**Affiliations:** 1 Mabwell (Shanghai) Bioscience Co., Ltd., Shanghai 201210, China.; 2 Beijing Kohnoor Science & Technology Co., Ltd., Beijing 102206, China.; 3 School of Life Sciences, Anhui University, Hefei 230022, China.

**Keywords:** Bispecific antibody, Immunotherapy, Phagocytosis, Therapeutic efficacy, Immunity.

## Abstract

**Background:** Even though PD-1/PD-L1 is an identified key “don't find me” signal to active adaptive immune system for cancer treatment, the overall response rate (ORR) for all cancer patients is still limited. Other effective therapeutic modalities to bridge the innate and adaptive immunity to improve ORR are urgently needed. Recently, CD47/SIRPα interaction is confirmed as a critical “don't eat me” signal to active innate immunity. However, the red blood cell (RBC) toxicity is the big concern for the development of CD47-based anti-cancer therapeutics.

**Methods:** Here, we report the development of a CD47/PD-L1 bispecific antibody 6MW3211 to block both PD-1/PD-L1 and CD47/SIRPα signals, and studied the effects of 6MW3211 on anti-tumor immune functions *in vitro* and *in vivo*. The pharmacokinetic and toxicity profiles of 6MW3211 were evaluated in GLP non-human primate (NHP) studies.

**Results:** The dual immune checkpoint inhibitory signaling blocker 6MW3211 shows high binding affinity to PD-L1 and low binding affinity to CD47. This inequivalent binding affinity design makes 6MW3211 preferentially bound to PD-L1 on tumor cells followed by disrupting the interaction of CD47/SIRPα. Complex structure determination and flow cytometry assay demonstrated that 6MW3211 has no binding to either human or rhesus monkey RBCs. 6MW3211 effectively blocked both PD-1/DP-L1 and CD47/SIRPα signaling and promoted macrophage phagocytosis of tumor cells. Potent therapeutic efficacies of 6MW3211 in three different mouse models were further observed. Moreover, 6MW3211 was demonstrated to have a fairly good safety profile in a GLP NHP study. In addition, multiplex fluorescent immunohistochemistry (mIHC) staining shows that PD-L1 and CD47 co-express on several different types of human tumor tissues.

**Conclusions:** These results support the development of 6MW3211 for the treatment of PD-L1 and CD47 double positive cancers.

## Introduction

Immune checkpoint inhibitory receptors are a kind of immunosuppressive molecules that express on immune cells and transduce inhibitory signaling to immune activation after engagement with their ligands expressing on cancer cells or other immune cells 1, 2. PD-1 is the most popular and well-studied immune checkpoint inhibitory receptor expressing on activated T cells 3. Tumor cells would express PD-L1 and use PD-1/PD-L1 signaling to inhibit T cell proliferation and activation to further evade immune supervision 4, 5. An increasing number of PD-1/PD-L1 signaling blocking antibodies have been approved for treatment of a variety of cancers 6. However, the ORR for all cancer patients is still below than 30%, and primary resistance and acquired resistance were reported for many patients in different clinical trials 7, 8. There are many reasons for these patients experienced primary resistance. The key reason is that not enough T cell infiltration, T cell proliferation and T cell activation was determined in tumor microenvironment. Novel modalities to active innate immunity for increasing tumor killing, tumor specific antigen presenting, tumor specific T cell proliferation and activation should benefit these patients with primary resistance to PD-1-based therapies.

CD47 is another “star” molecule in immunotherapy field, expressing on tumor cells and delivering a “don't eat me signal” to macrophage to inhibit the innate phagocytosis once binding to its ligand signal-regulatory protein alpha (SIRPα) 9, 10. Disrupting antibodies targeting both CD47 or SIRPα have been confirmed their therapeutic efficacy in both pre-clinical studies and clinical trials 11. Several publications showed that knocking out CD47 on tumor cells could enhance macrophage phagocytosis, antigen presentation and T cell infiltration in different animal models 12. All these information supports the development of CD47-based therapies for cancer treatment. However, CD47 also expresses on a wild range of normal cells, especially on RBCs to inhibit the clearance by macrophages 13, 14. Severe hematologic toxicity was observed in almost all clinical trials for Magrolimab (Hu5F9, developed by Forty-Seven, Inc.), and hampered its further clinical development 13. Recently, two glycosylation sites on CD47 in the interface of CD47 with SIRPα were identified, and several SIRPα mutants with different binding ability to CD47 expressing on human RBCs and tumor cells were reported, which makes it possible to discover antibodies specifically binding to tumors cells with no cross-reactivity with CD47 on RBCs 15, 16. Thus, Development of anti-CD47 based therapeutics with no or limited binding to RBCs is an attractive direction for both hematological malignancies and solid tumors treatment.

Currently, the majority of cancer immunotherapies activate either innate immunity or adaptive immunity. Theoretically, enhanced therapeutic efficacy would be achieved if cancer therapies could stimulate both innate and adaptive immunity. As described above that PD-1/PD-L1 and CD47/SIRPα signals mainly suppress adaptive and innate immune responses, respectively. Simultaneously targeting these two pathways provides promising solutions for cancer treatment. Several studies have demonstrated the enhanced therapeutic efficacy of dual blockade of CD47/SIRPα and PD-1/PD-L1 signaling in the treatment of different types of cancers 17.

In this research, we generated a CD47/PD-L1 dual-targeting bispecific antibody 6MW3211 using knobs-into-holes (KIH) technology with common light chain design. 6MW3211 was designed with high binding affinity to hPD-L1 and low affinity to hCD47, which allows 6MW3211 to preferentially bind to hPD-L1 expressing tumor cells followed by CD47/SIRPα signaling disrupting. Potent therapeutic efficacy of 6MW3211 was observed in different mouse models. Moreover, 6MW3211 showed no binding to either human or rhesus monkey RBCs *in vitro*, and exhibited quite good pharmacokinetic and safety profile in GLP non-human primate (NHP) studies. In addition, using mIHC staining, PD-L1 and CD47 were demonstrated co-expression on several types of tumor cells. These data pave the way for development of 6MW3211 for further clinical trials.

## Materials and methods

### Cell lines

All these cell lines used for this study were purchased from ATCC. PD-L1 overexpressed HEK293-GFP (HEK293-hPD-L1-GFP), PD-L1 and CD47 co-overexpressed MC38 (MC38-hPD-L1/hCD47), PD-L1 overexpressed Raji (Raji-hPD-L1), Raji-luc and Raji-hPD-L1-luc overexpressed cell lines were prepared by lentivirus infection technology according to standard protocols. All cell lines were maintained in RPMI 1640 (Cat: L210KJ, BasalMedia) supplemented with 10% FBS (Cat: FSP500, EXCELLBIO). Cell lines with passage 5 to 25 were used for* in vitro* experiments. No additional mycoplasma or authentication tests were performed.

### Bispecific antibody generation and characterization

For anti-PD-L1 and anti-CD47 antibody generation, balb/c mice were immunized with human PD-L1 or human CD47 recombinant protein prepared by Beijing Kohnoor Science & Technology Co., Ltd. After that, hybridomas were generated and the supernatants were assayed for hPD-L1 or hCD47 binding by ELISA and FACS. The positive samples were then subcloned followed by variable region (VH and VL) genes sequencing. Individual recombinant antibody was expressed in HEK-293 cells and purified with protein A resin. Purified antibodies were then assessed in different functional assays, and the top 10 candidates were humanized using CDR grafting strategy. After several rounds of heavy chains and light chains combination, screening, engineering and optimization, anti-CD47 and anti-PD-L1 antibodies with common light chain were obtained.

PD-L1/CD47 bispecific antibody 6MW3211 was generated as full-length human IgG4 subtype using the knobs-into-holes (KIH) technology as reported 18. The knob arm used anti-CD47 heavy chain, and the hole arm used anti-PD-L1 heavy chain. 6MW3211 was expressed either in HEK293 cells or CHO cells. After production and purification, mass spectrometry and exclusion chromatography were used to evaluate the half-antibody and antibody aggregates. The endotoxin content of different bathes of 6MW3211 was below 0.5 EU/mg.

### Kinetic analysis for the binding affinity of 6MW3211

The binding affinity of 6MW3211 to hPD-L1 and hCD47 recombinant proteins was determined at 25 ℃ by BIAcore T200 system using CM5 biosensor chips (Cytiva). For all measurements, 1-ethyl-3-(3-dimethylaminopropyl) carbodiimide hydrochloride (200 mM) and N-hydroxysuccinimide (50 mM) were mixed 1:1 and injected into CM5 sensor chip. Anti-human IgG Fc antibody was diluted to 20 µg/mL with sodium acetate (pH 4.5) then coupled to the chip. The remaining activation sites were blocked by injection of 1 Mol ethanolamine (pH 8.5), the coupled chips were equilibrated in HBS-EP+ buffer (10 µL/min). All proteins used for this assay were related buffer exchanged in advance. The blank channel of the chip was used as the negative control. 6MW3211 was captured on the chip first. Gradient concentrations of hPD-L1 (from 20 nM to 0.3125 nM with 2-fold dilution) or hCD47 recombinant proteins (from 2000 nM to 31.25 nM with 2-fold dilution) were injected and then flowed over the chip surface. The sensor chip was regenerated with Gly-HCl (pH 1.5) after each cycle. The 1:1 (Rmax Local fit) fitting model with BIA evaluation software was used to calculate the affinity of tested antibodies. Meanwhile, simultaneously binding of 6MW3211 to hPD-L1 and hCD47 was measured by Octet RED96 system. 6MW3211 (4 μg/mL) was captured by AHC biosensors followed by flowing PD-L1 (200 nM) for 240 s and CD47 (60 nM) for 300 s. The reversed experiment was performed by exchanging the order of the flowing antigen PD-L1 and CD47.

### Flow cytometry assay

The binding of 6MW3211 to HEK293-hPD-L1-GFP and MDA-MB-231cells were performed by FACS. Cells were collected and washed twice with 1 × PBS, followed by blocking with 10% FBS (Cat: FSP500, EXCELLBIO) in RPMI 1640 for 20 min at room temperature. 3-fold serially diluted 6MW3211 starting from 66 nM was added to cells (2 × 10^5^ cells/sample in 100 µL) and incubated on ice for 45 min. After washing the cells for two times with 1 × PBS, Goat Anti-human IgG Fc-FITC antibody (Cat: F9512, Sigma) (1: 200 dilution) was added and incubated on ice in dark for 60 min, followed by flow cytometry checking with CytoFLEX (Beckman Coulter).

### Generation of Fab/CD47 complex

Recombinant human CD47 extracellular domain protein (SN: NP_942088.1, 19 aa - 134 aa) was expressed in CHO cells and purified by affinity chromatography. 6MW3211-CD47 Fab was expressed in HEK293 cells and purified with Protein G affinity chromatography (Cat: 17-5248-01, Cytiva). These purified recombinant proteins were buffer exchanged into PBS using Vivacon 500 concentrator (Cat: VS0122, Sartorius Stedim). SDS-PAGE and SEC-HPLC were then used to check the purity and size of 6MW3211-CD47 Fab. Then, hCD47 recombinant protein and 6MW3211-CD47 Fab were mixed in with the molar ratio of 3:1 in 20 mM Tris with 150 mM NaCl solution. After incubation at room temperature for 5 h, the 6MW3211-CD47 Fab/hCD47 complex was obtained, and then concentrated to the concentration of 15 mg/mL. The quality of the antigen-antibody complex was tested using SEC-HPLC and SDS-PAGE before it was used for crystal preparation.

### Crystallization, data collection, structure determination, and analysis

The crystals of 6MW32-CD47 Fab/hCD47 complex were grown in a buffer composed of 2.2 M DL-malic acid (pH 5.5) using the sitting-drop vapor-diffusion method at 16 °C. The X-ray diffraction data of 6MW32-CD47 Fab/hCD47 complex were generated at beamline BL19U1 19 at Shanghai Synchrotron Radiation Facility with the wavelength of 0.9785 Å and processed with the HKL3000 package 20. 6MW32-CD47 Fab/hCD47 complex structure was then determined by molecular replacement method by PHASER 21, using the hCD47 (PDB code: 5TZU) and MW317 Fab (PDB code: 6JJP) structures as the search models. The model was rebuilt with COOT 22 and refined with PHENIX 23. All related data, such as the structure factors and atomic coordinates were deposited into the Protein Data Bank (PDB) under the accession number of 7XJF. Detailed information for the data collection and structure determination are shown in Table S1.

### *In vitro* blocking assay

The PD-1/PD-L1 signaling blocking assay in reporter system was performed according to the manufacturer's instruction (NIFDC). In short, CHO-PD-L1-CD3L cells were collected and seeded at the density of 4 × 10^5^ cells/well (100 μL) in 96-well plates. After culturing for 24 h, the supernatant was discarded and the CHO-PD-L1-CD3L cells were incubated with tested antibody and Jurkat-PD1-NFAT cells (6 × 10^4^/well) in 50 μL assay buffer (5% FBS in 95% RPMI-1640) for 6 h in a 37 °C incubator with 5% CO_2_. 50 μL Bio-Lite Reagent (Cat: DD1201-02, Vazyme) was added to each well followed by incubating in dark for 5 min. After that, the luminescent signal was measured by INFINITEF Plex.

For the CD47/SIRPa interaction blocking assay, recombinant hCD47 (0.3 µg/mL) and hPD-L1 (2 µg/mL) were co-coated on a 96-well ELISA plate in 100 µL at 4 °C overnight. After washing and blocking, tested antibodies (concentrations from 1320 nM to 0.067 nM, 100 μL/well) were added to the plates and incubated for 30 min at 37 °C. Then the biotin-hSIRPα-mFc (concentration: 6.6 nM, 100 μL/well) was added into the plates followed by incubation at 37 °C for 2 h. After washing the plates, secondary Ab HRP-Streptavidin (Cat: RABHRP3, Sigma) was added and incubated for 20 min. TMB substrate was used for color development and the absorbance singal at 450 nm was measured.

### *In vitro* phagocytosis assay

For the *in vitro* macrophage phagocytosis of Raji or Raji-hPD-L1 cells mediated by 6MW3211, macrophages derived from hCD47/hSIRPa transgenic mice were used. Mice were injected intraperitoneally with starch broth (Cat: 10021318, Ourchem) at 1.5 mL/animal. After 48 h, the peritoneal macrophages were isolated and inoculated in a 12-well plate about 25% confluent. After 24 h, macrophages were starved with serum-free medium for 2 h. Target cells (Raji or Raji-hPD-L1) were stained with calcein (20 μM) (Cat: C326, DojinDo). Antibodies (132 nM) were pre-incubated with stained cells at 37 ℃ for 1 h. Then, the mixtures were incubated with macrophages at 37 ℃ for 2 h, the E/T ratio was 1:5. After washing out unphagocytosed target cells, macrophages were harvested and stained with F4/80-APC (Cat: E-AB-F0995E, Elabscience). The mice F4/80 positive cells population (murine macrophages, APC+) were selected for analysis by flow cytometry. The population of double positive cells (green fluorescence+ and APC+) indicated the phagocytosed macrophages. The phagocytosis index (%) was calculated by double positive cells divided by APC single positive cells.

### Binding of 6MW3211 to human and rhesus monkey red blood cells

Anti-coagulant peripheral blood from human and rhesus monkeys were collected, and the red blood cells were extracted with human erythrocyte extraction kit (Cat: RBC2014TBD, Tbdscience) and monkey erythrocyte extraction kit (Cat: RBCHY2012MK, Tbdscience). Red blood cells were adjusted to 5 × 10^6^ cells/mL in RPMI 1640 complete medium. Then, cells were added into a U-shaped-bottom cell culture plate at 100 µL/well. Five-fold serially diluted antibodies starting from 660 nM were added to cells and incubated at 4 ℃ for 1 h. After that, cells were washed and added with PE-babbled goat anti-human IgG Fc (Cat: F9512, Sigma) for 45 min. Cells were washed for 3 times and analyzed with flow cytometry.

### Tissue distribution of 6MW3211

6MW3211 was radiolabeled with ^89^Zr (Perkin Elmer, Inc.) using the protocol previously reported 24, 25. Six B-hPD-L1/hCD47/hSIRPα triple transgenic mice bearing MC38-hPD-L1/hCD47 tumor were used to evaluate tumor uptake and tissue distribution. After a single injection of ^89^Zr labeled 6MW3211 (20 mg/kg, 200 μCi/mouse), PET/CT scans of 10 to 30 min were performed at 2, 24, 48, 72, 96, 120, 168 and 336 h post dose. Images were taken and then reconstructed following the acquisition of the animal scanning data. Image analysis software PMOD was used to depict the regions of interests (ROIs) for tumor and normal tissues. The percentages of injected dose per gram (%ID/g) were then calculated, and the radiopharmaceutical concentrations in different tissues of each animal were calculated.

### *In vivo* anti-tumor efficacy with mouse models

All animal studies were conducted in compliance with Yicon's guidelines for the care and use of laboratory animals and all protocols were approved by the Institutional Animal Care and Use Committee (IACUC) of Yicon (Beijing) BioMedical Technology Inc.

For systemic Raji-luc or Raji-hPD-L1-luc human lymphoma xenograft model, tumor cells Raji-luc or Raji-hPD-L1-luc cells were suspended in PBS to 5 × 10^6^/mL and inoculated intravenously in each NCG mouse (GemPharmatech Co., Ltd) with 100 µL/mouse. After tumor cells inoculation for 3 days, mice were randomized based on bioluminescence imaging. Different doses of 6MW3211 (0.04, 0.2, 1.0 or 5.0 mg/kg) or control antibody (5.0 mg/kg) were administrated twice weekly by intraperitoneal injection. The mice were imaged weekly with an *In Vivo* Imaging System (IVIS Lumina Series III, PerkinElmer), using average radiance of bioluminescence signal to indicated tumor burden.

For MC38-hPD-L1/hCD47 murine colon carcinoma model, MC38-hPD-L1/hCD47 cell line was constructed by knocking out mouse PD-L1 and CD47 gene and knocking in human PD-L1 and CD47 gene. B-hPD-L1/hCD47/hSIRPα triple transgenic mice were subcutaneously injected with MC38-hPD-L1/hCD47 cells (2 × 10^5^ cells) in 0.1 mL PBS in the right front flank for tumor development. Forty tumor-bearing mice with mean tumor size of approximately 88 mm^3^ were selected and randomly enrolled into five groups. Different doses of 6MW3211 (0.5, 2.0, 8.0 or 20.0 mg/kg), and control antibody (20.0 mg/kg) were administrated twice weekly by intraperitoneal injection. Tumor size was measured twice weekly. Tumor growth inhibition rate (TGI) for each mouse was calculated accordingly.

### PK and toxicity study of 6MW3211

All rhesus monkey-related experiments were conducted at JOINN Laboratories in accordance with standard operating procedure and the related protocols were complied with relevant ethical regulations.

For pharmacokinetic study, a total of 18 naïve rhesus monkeys randomly divided by 3 groups (3 animals/sex/group) were enrolled for this study and administered with 6MW3211 at 3, 10 and 30 mg/kg with a single intravenous infusion. The blood sample for concentration analyses were collected at pre-dose, and 15 min, 2 h, 4 h, 8 h, 24 h, 48 h, 96 h, 120 h, 168 h, 240 h, 336 h, 408 h, 504 h and 672 h post dose. Serum concentrations of 6MW3211 were then determined with a validated ELISA method.

GLP-compliant 4-week repeated dose toxicity study was conducted using a total of 40 rhesus monkeys (5 animals/sex/group, QW × 5) at dose of 20, 60 and 200 mg/kg, following a 4-week recovery period. Evaluations and their related parameters included clinical observations, food consumption, safety pharmacology, gross pathology, clinical pathology, immunotyping, ADA analysis, immunoglobulins, toxicokinetics and histopathology.

### Multiplex fluorescent immunohistochemistry (mIHC) staining

Multiplex staining of hPD-L1 and hCD47 co-expression on cancer tissues was performed using 4-color kit (WiSee Biotechnology), according to manufacturer's instruction. Three primary antibodies are anti-CD47 (Cat: ab218810, diluted 1:500, Abcam), anti-PD-L1 (Cat: 13684, diluted 1:400, CST) and anti-PanCK (Cat: CM351507, diluted 1:200, Gene Tech). After applied different primary antibodies (anti-CD47, anti-PD-L1 and anti-PanCK, sequentially), the secondary antibody (HRP conjugated) was added and incubated, followed by tyramide signal amplification (Cat: M-D110051, WiSee Biotechnology). After all antigens being labeled with different antibodies, DAPI (Cat: D1306, ThermoFisher) was used for nuclei staining. Pannoramic MIDI imaging system (3D HISTECH) was then used for scanning the stained slides. The number of target cells were analyzed by HALO software (Indica Labs).

## Statistical analysis

Statistical analysis was used for *in vitro* functional assays and *in vivo* experiments. Data are shown as mean ± SEM. Statistical analyses were performed with GraphPad 7.0 software. The animal survival was using Kaplan-Meier analysis and Log Rank test. The differences between groups were analyzed for significance using the One-Way Analysis of Variance (One-Way ANOVA) followed by multiple comparison test and P < 0.05 was considered as statistically significant.

## Results

### Design and characterization of 6MW3211

**Figure 1:**
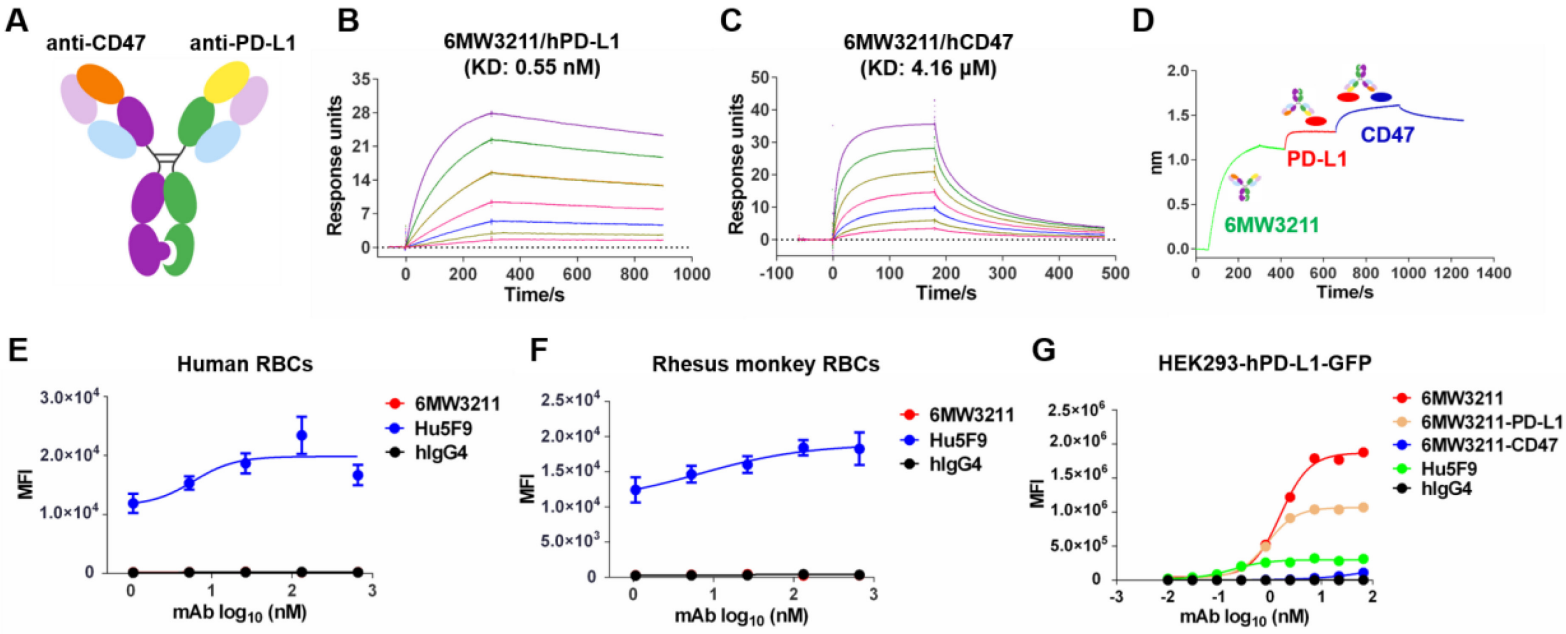
** Generation and characterization of 6MW3211. (A)** Schematic diagram of the CD47/PD-L1 bispecific antibody 6MW3211. **(B)** Binding affinity measurement of 6MW3211 to hPD-L1 was performed by BIAcore T200 system. The KD of 6MW3211 to hPD-L1 was 0.55nM. **(C)** Binding affinity measurement of 6MW3211 to hCD47 was performed by BIAcore S200 system. The KD of 6MW3211 to hCD47 was 4.16 μM. **(D)** Simultaneously binding of 6MW3211 to hPD-L1 and hCD47 measured by Octet RED96 system. Binding of 6MW3211 to human **(E)** and rhesus monkey **(F)** RBCs. Red blood cells were adjusted to 5 × 10^6^ cells/mL, and added into a U-shaped-bottom cell culture plate at 100 µL/well. 5-fold serially diluted antibodies starting from 660 nM were added to cells. After that, cells were washed and added with PE-babbled goat anti-human IgG Fc and analyzed with flow cytometry. **(G)** Binding of 6MW3211 to hPD-L1 overexpressing HEK293-GFP (HEK293-hPD-L1-GFP) cells.

6MW3211 is a humanized bispecific antibody with an IgG4 format using knobs-in-holes (KIH) technology and the common light chain structure (Figure 1A). The knob arm is anti-CD47 heavy chain, and the hole arm is anti-PD-L1 heavy chain. Each arm was discovered independently using traditional mouse hybridoma strategy followed by characterization and humanization. After several rounds of engineering and optimization, the final humanized CD47/PD-L1 bispecific antibody 6MW3211 was obtained with high binding ability to human PD-L1 (hPD-L1) and relatively low binding ability to human CD47 (hCD47). Binding affinity of each arm of 6MW3211 was further measured by BIAcore based on surface plasmon resonance (SPR). The KD of 6MW3211 to hPD-L1 and hCD47 are 0.55 nM and 4.16 μM, respectively (Figure 1B-C). The high affinity of 6MW3211 to hPD-L1 and low affinity to hCD47 was supposed to navigate 6MW3211 to hPD-L1 expressing tumor cells followed by disrupting CD47/SIRPα interaction. 6MW3211 was further demonstrated to bind to both hCD47 and hPD-L1 simultaneously (Figure 1D and Figure S1).

As CD47 is widely expressed on circulating red blood cells (RBCs) and other hemopoietic cells, the potential cytotoxicity, especially the cytotoxicity for RBCs is the major concern for development of anti-CD47 based therapeutics. During the whole process of anti-CD47 antibody screening and optimization, binding to CD47 specifically expressed on tumor cells, but not RBCs was set as the first criteria. The binding of 6MW3211 to human and rhesus monkey RBCs was evaluated by flow cytometry. As shown in Figure 1E and 1F, 6MW3211 exhibited no binding to either human or rhesus monkey RBCs, while strong binding signals were detected for anti-CD47 control antibody Hu5F9 (Forty-Seven, Inc.), indicating that 6MW3211 may have a good safety profile in non-clinical and clinical studies. Further, cell-based binding experiments with HEK293-GFP cells over-expressing hPD-L1 (HEK293-hPD-L1-GFP) was performed by flow cytometry. The EC_50_ value of 6MW3211 was 1.58 nM, which is slightly lower than parental bivalent anti-PD-L1 antibody 6MW3211-PD-L1 (0.88 nM). As expected, the mean fluorescence intensity (MFI) in the platform for monovalent 6MW3211 is about two-fold higher than that of bivalent 6MW3211-PD-L1(Figure 1G).

### Determination of the complex structure of 6MW3211-CD47 Fab with hCD47

**Figure 2:**
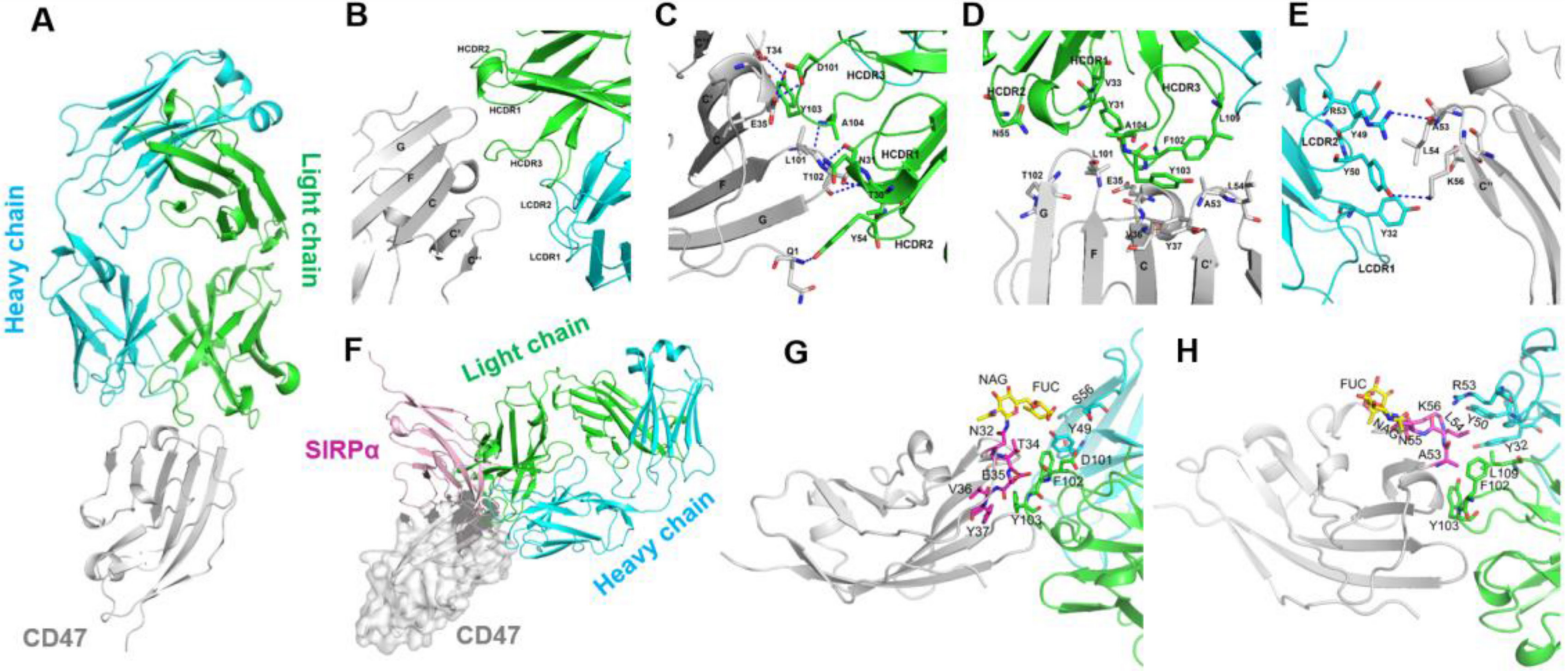
** Decoding the interaction of 6MW3211-CD47 Fab with hCD47. (A)** Overall structure of 6MW3211-CD47 Fab in complex with hCD47. **(B)** Interaction of CDR regions of Heavy and Light chains of 6MW3211-CD47 Fab with β-strand C, β-strand C', C'C“ loop and FG loop of CD47. **(C)** Hydrogen bonding interaction of Heavy chain CDR1 and CDR3 with CD47. **(D)** Hydrophobic interaction of Heavy chain CDR1 and CDR3 with CD47. **(E)** Interaction of Light chain CDR1 and CDR2 with CD47. Green and sky blue represent the Heavy and Light chains of 6MW3211-CD47 Fab, respectively, and gray represents CD47. The residues involved in the interaction are shown in a rod model, and the hydrogen bonding is indicated by blue dashed lines. **(F)** Superimposing CD47 of CD47/SIRPα complex (PDB code: 2JJS) with CD47 of 6MW3211-CD47 Fab/CD47 complex (PDB code: 7XJF). Grey represents CD47, pink represents SIRPα. **(G)** The detail interaction of 6MW3211-CD47 Fab with hCD47 nearby residue N32. **(H)** The detail interaction of 6MW3211-CD47 Fab with hCD47 nearby residue N55.

To explore the molecular mechanism of tumor cell binding selectivity of 6MW3211-CD47, the crystal structure of 6MW3211-CD47 Fab in complex with hCD47 was solved at a resolution of 2.6 Å (PDB code: 7XJF). The complex structure includes hCD47 extracellular domain (1-116 aa), 6MW3211-CD47 Fab heavy chain (1-226 aa), and light chain (1-213 aa). The hCD47 has a glycosylated structure at sites N16, N32, N55, and N93. The elucidated crystal structure contains one CD47 molecule and one 6MW3211-CD47 Fab, forming a 1:1 complex structure (Figure 2A). The interaction surface between CD47 and 6MW3211-CD47 Fab had an area of approximately 637 Å^2^, and the interaction mainly involved β-strand C, CC' loop, β-strand C' and FG loop of CD47. Heavy chain HCDR1, HCDR2, HCDR3 and light chain LCDR1 and LCDR2 were involved in the recognition of CD47. Interaction modes include van der Waals forces, hydrogen bonding and hydrophobic interactions. The amino acid residues for specific interactions are shown in Figure 2B to 2E and Table S2.

By comparison to the complex structure of CD47/SIRPα (PDB code: 2JJS), most of the binding surface of 6MW3211-CD47 Fab to CD47 coincides with the binding surface of SIRPα, suggesting that 6MW3211-CD47 Fab competes against SIRPα for CD47 binding (Figure 2F and Figure S2). Further crystal structure analysis showed that the N-linked glycol chains of N32 and N55 of hCD47 contributed to the binding of 6MW3211-CD47 Fab (Figure 2G-H), which indicates that the selectivity of 6MW3211-CD47 binding to CD47 expressing on tumor cells may be due to the difference of N-linked glycosylation at position N32 and N55 between tumor cells and RBCs.

### 6MW3211 blocking both CD47/SIRPα and PD-1/PD-L1 signaling and promoting macrophage phagocytosis *in vitro*

**Figure 3:**
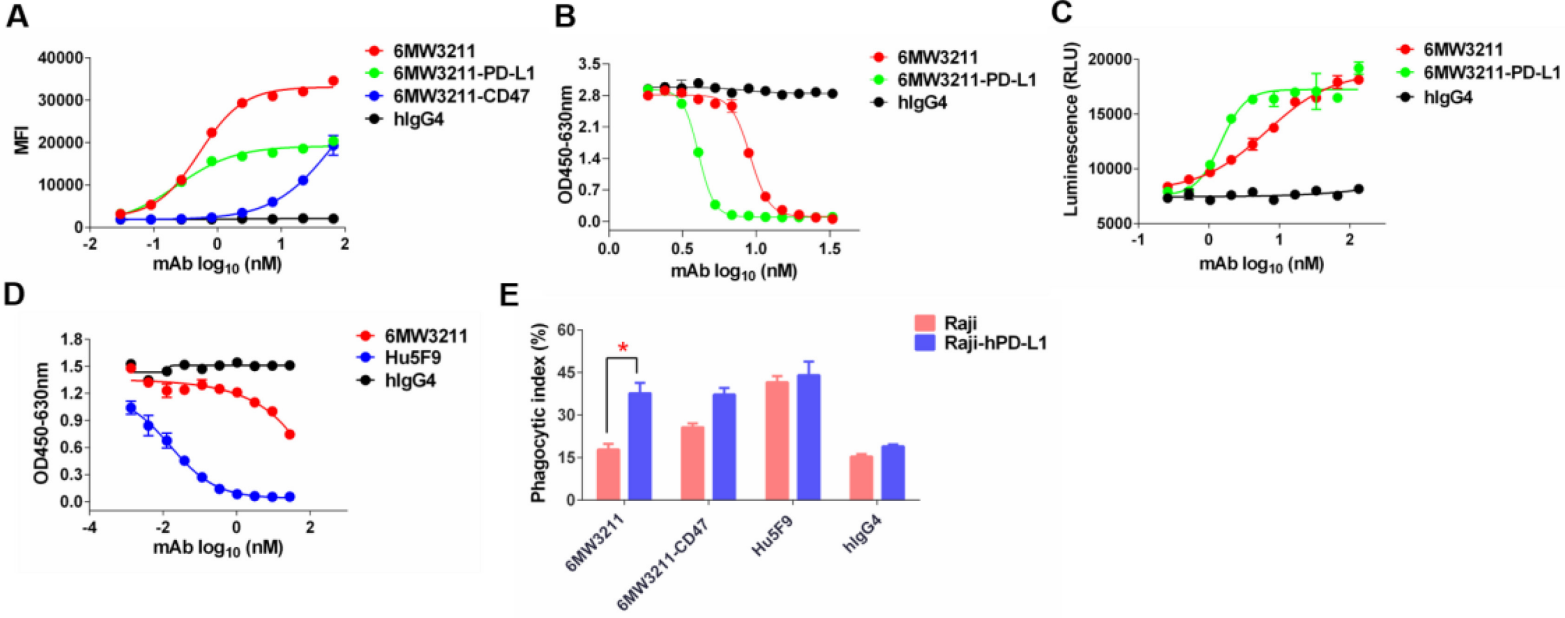
** 6MW3211 disrupting both CD47/SIRPα and PD-1/PD-L1 signals and promoting macrophage phagocytosis of tumor cells. (A)** 6MW3211 binding to MDA-MB-231 tumor cells. 6MW3211 exhibited quite strong binding to MDA-MB-231 cells, with EC_50_ of 0.53 nM, similar to the parental anti-PD-L1 antibody. **(B)** 6MW3211 blocking the interaction of PD-1 and PD-L1 performed in ELISA. **(C)** 6MW3211 disrupting the PD-1/PD-L1 signaling in reporter system. **(D)** 6MW3211 blocking the interaction of CD47/SIRPα performed in ELISA. **(E)** Macrophage phagocytosis of Raji and Raji-hPD-L1 cells mediated by 6MW3211. The concentrations for all antibodies used in this assay was 132 nM. Significance was calculated by two-way ANOVA with Sidak's multiple comparisons test. “*” means p < 0.05.

The major purpose for the design of 6MW3211 is to obtain therapeutic benefit by selectively blocking the interaction of CD47 and SIRPα on PD-L1 and CD47 double positive tumor cells, but not binding to CD47 expressing on RBCs and other normal cells. To assess the binding ability of 6MW3211 to CD47 and PD-L1 double positive human cancer cells, MDA-MB-231 was used for flow cytometry analysis, and the parental anti-CD47 and anti-PD-L1 bivalent antibodies were used as control. As shown in Figure 3A, 6MW3211 exhibited quite strong binding to MDA-MB-231 cells, with EC_50_ of 0.53 nM, comparable to its parental anti-PD-L1 antibody. The disrupting activity of 6MW321 on PD-1/PD-L1 interaction was evaluated by both ELISA and cell-based reporter system. 6MW3211 efficiently disrupted the interaction of PD-1/PD-L1 in ELISA. As expected, the IC_50_ of 6MW3211 was greater than that of parental bivalent 6MW3211-PD-L1. In the high concentration condition, both 6MW3211 and 6MW3211-PD-L1 completely blocked the interaction of PD-1/PD-L1 (Figure 3B). 6MW3211 also attenuated the inhibitory effect of PD-1/PD-L1 on CD3L/TCR signaling in reporter system, indicating that 6MW3211 could positively regulate T cells by PD-1/PD-L1 blocking activity (Figure 3C).

The disrupting of CD47/SIRPα interaction by 6MW3211 was also determined by ELISA. Based on the high affinity to hPD-L1 and low affinity to hCD47 design, 6MW3211 efficiently blocked the interaction of CD47/SIRPα when co-coated with hPD-L1 in ELISA (Figure 3D and Figure S3). This result confirmed that once anti-PD-L1 arm of 6MW3211 binding to hPD-L1, the anti-CD47 arm could block the interaction of CD47/SIRPα. To further evaluate whether 6MW3211 could induce the macrophage phagocytosis of tumor cells or not, wild-type Raji cells with no or low hPD-L1 expression, and Raji cells with hPD-L1 overexpression (Raji-hPD-L1) were used for *in vitro* phagocytosis assays. The result revealed that, 6MW3211 increased the phagocytosis of Raji-hPD-L1 cells by peritoneal macrophages from hCD47 and hSIRPα double transgenic mice. Notably, the phagocytosis index of Raji-hPD-L1 cells mediated by 6MW3211 was significantly higher than that of Raji cells, which further confirmed that anti-PD-L1 arm of 6MW3211 could enhance the blockade of CD47/SIRPα by anti-CD47 arm (Figure 3E and Figure S4). These results indicated that strong PD-L1 binding can boost the phagocytosis-enhancing effect, which is in line with the target binding and blockade studies.

Together, these results demonstrated that 6MW3211 could effectively block the PD-1/PD-L1 inhibitory signaling and promote macrophage phagocytosis of hCD47 and hPD-L1 double positive tumor cells.

### Therapeutic effect of 6MW3211 *in vivo*

**Figure 4:**
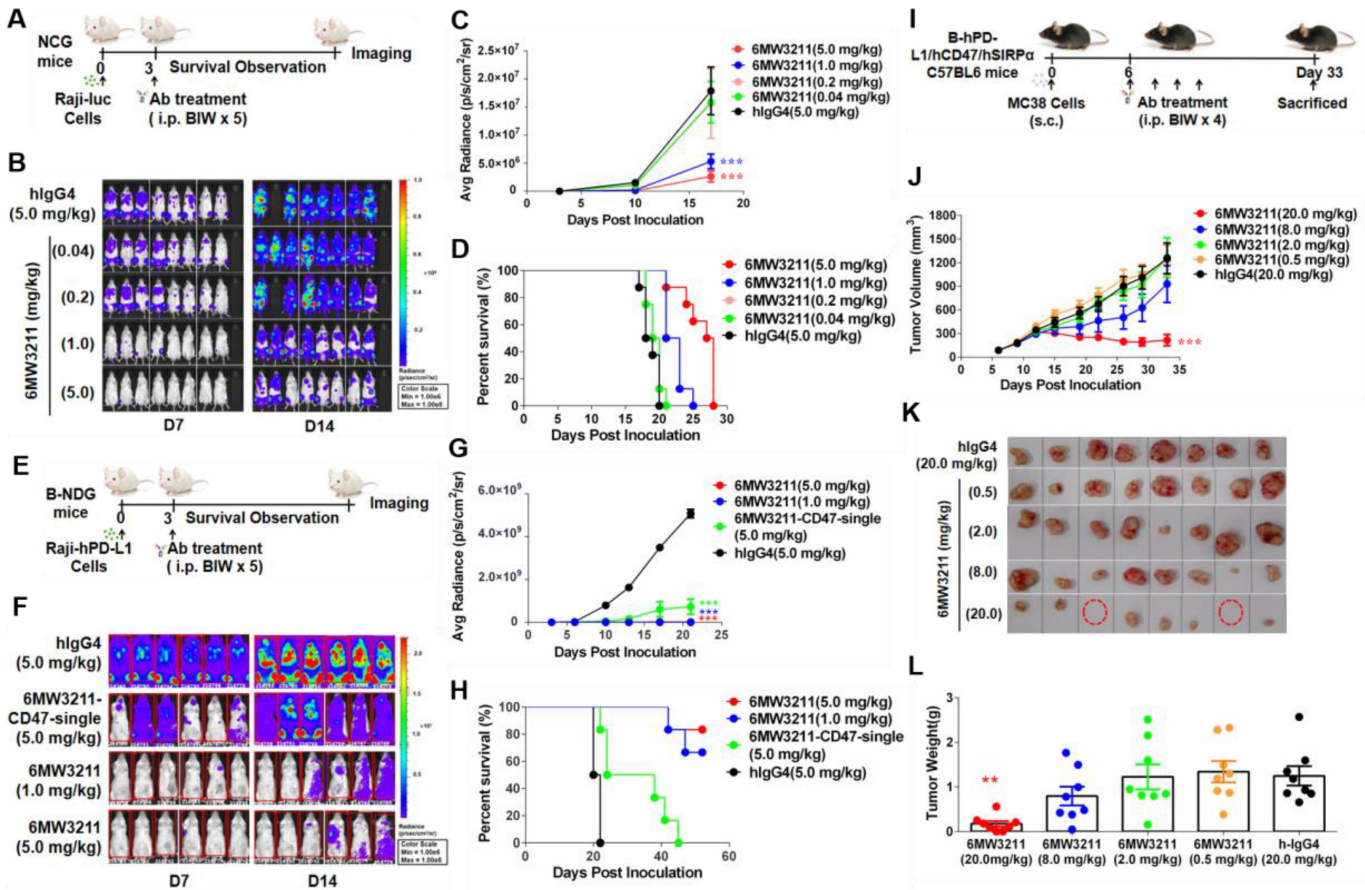
** Anti-tumor activity of 6MW3211 in different mouse models. (A)** The schematic diagram of the study design of Raji-luc/NCG model. 6MW3211 with different doses (0.04, 0.2, 1.0 and 5.0 mg/kg) or control antibody (hIgG4, 5.0 mg/kg) were administrated 3 days after Raji-luc cells injection. **(B)** Bioluminescence imaging of mice treated with 6MW3211 or control IgG. **(C)** Quantified bioluminescence signals for mice treated with 6MW3211 or control IgG. P value was labeled. **(D)** Kaplan-Meier survival curves of the Raji-luc cells in mice treated with the indicated doses of 6MW3211. **(E)** The schematic diagram of the study design of Raji-hPD-L1-luc/B-NDG model. 6MW3211 (1.0 or 5.0 mg/kg), the monovalent parental anti-CD47 arm (6MW3211-CD47-single) or control antibody (5.0 mg/kg) were administrated 3 days after Raji-hPD-L1-luc injection. **(F)** Bioluminescence imaging of mice treated with 6MW3211 or control IgG. **(G)** Quantified bioluminescence signals for mice treated with 6MW3211 or control IgG. P value was labeled. **(H)** Kaplan-Meier survival curves of the Raji-hPD-L1-luc cells in mice treated with the indicated doses of 6MW3211. **(I)** The schematic diagram of the study design of MC38-hPD-L1/hCD47 (B-hPD-L1/hCD47/hSIRPα) model. 6MW3211 with different doses (0.5, 2.0, 8.0 and 20.0 mg/kg) or control antibody (hIgG4, 20.0 mg/kg) were injected for 4 times. **(J)** Growth curves of MC38-hPD-L1/hCD47 tumors in B-hPD-L1/hCD47/hSIRPα mice treated with indicated doses of 6MW3211. **(K)** Photos of excised tumors treated with 6MW3211. **(L)** Tumor weight on day 33. The data reported as a Mean ± SEM and the significance was calculated by two-way ANOVA with Tukey's multiple comparison test compared with hIgG4. “*” means p < 0.05; “**” means p < 0.01 and “***” means p < 0.001.

Anti-tumor efficacy of 6MW3211 was evaluated in three different mouse models. In the systemic Raji-luc human lymphoma xenograft model (NCG mouse), 6MW3211 with different doses (0.04, 0.2, 1.0 and 5.0 mg/kg) or control antibody (hIgG4, 5.0 mg/kg) were administrated 3 days after Raji-luc cells injection (Figure 4A). The mice in all treatment groups had rather stable body weights without any other apparent abnormality right after each dosing after randomization, the bioluminescence signals in the mice increased as tumors developed after randomization. 6MW3211 inhibited tumor progression and prolonged the overall survival of treated mice in a dose dependent manner (Figure 4B-D). As Raji-luc cells expressing very low level of hPD-L1, the anti-CD47 arm of 6MW3211 may play a critical role in anti-tumor activity in this Raji-luc xenograft model. These results indicated that 6MW3211 could effectively restore the phagocytosis of CD47-expressing cancer cells by blocking the inhibitory signal triggered by the interaction between mSIRPα expressed on mouse macrophages and hCD47 expressed on tumor cells.

Raji-hPD-L1-luc human lymphoma xenograft model (B-NDG mouse) was further used to assess whether anti-PD-L1 arm could enhance the anti-tumor activity of 6MW3211 or not. In this model, 6MW3211 (1.0 or 5.0 mg/kg), the monovalent parental anti-CD47 arm (6MW3211-CD47-single) or control antibody (5.0 mg/kg) were administrated 3 days after Raji-hPD-L1-luc injection (Figure 4E). In consistent with the *in vitro* phagocytosis data, co-engagement of hPD-L1 could strongly enhance the anti-tumor activity of 6MW3211 (Figure 4F). In the Raji-hPD-L1-luc model, 6MW3211 effectively inhibited tumor growth and prolonged about two-fold of overall survival time than that in Raji-luc model (Figure 4F-H).

Moreover, MC38-hPD-L1/hCD47 in humanized B-hPD-L1/hCD47/hSIRPα triple transgenic mouse model was used to evaluate the anti-tumor efficacy of 6MW3211 (Figure 4I). No abnormal body weight changes or signs of toxicity was observed throughout the study. 6MW3211 effectively inhibited tumor cell growth, especially in the 20 mg/kg treated group, in which two mice were cured (Figure 4J-L). We also evaluated the immune cells infiltration in tumor tissues, especially T cells, after treatment with 6MW3211. The proportion of lymphocytes (CD45^+^) and CD8^+^ T cells was increased, which indicated that 6MW3211 could induce the immune cells infiltration and activation in tumor microenvironment (Figure S5). All these data confirmed 6MW3211 as a potent therapy to inhibit tumor progression in *vivo*.

### The organ distribution of 6MW3211 in tumor bearing mice

**Figure 5:**
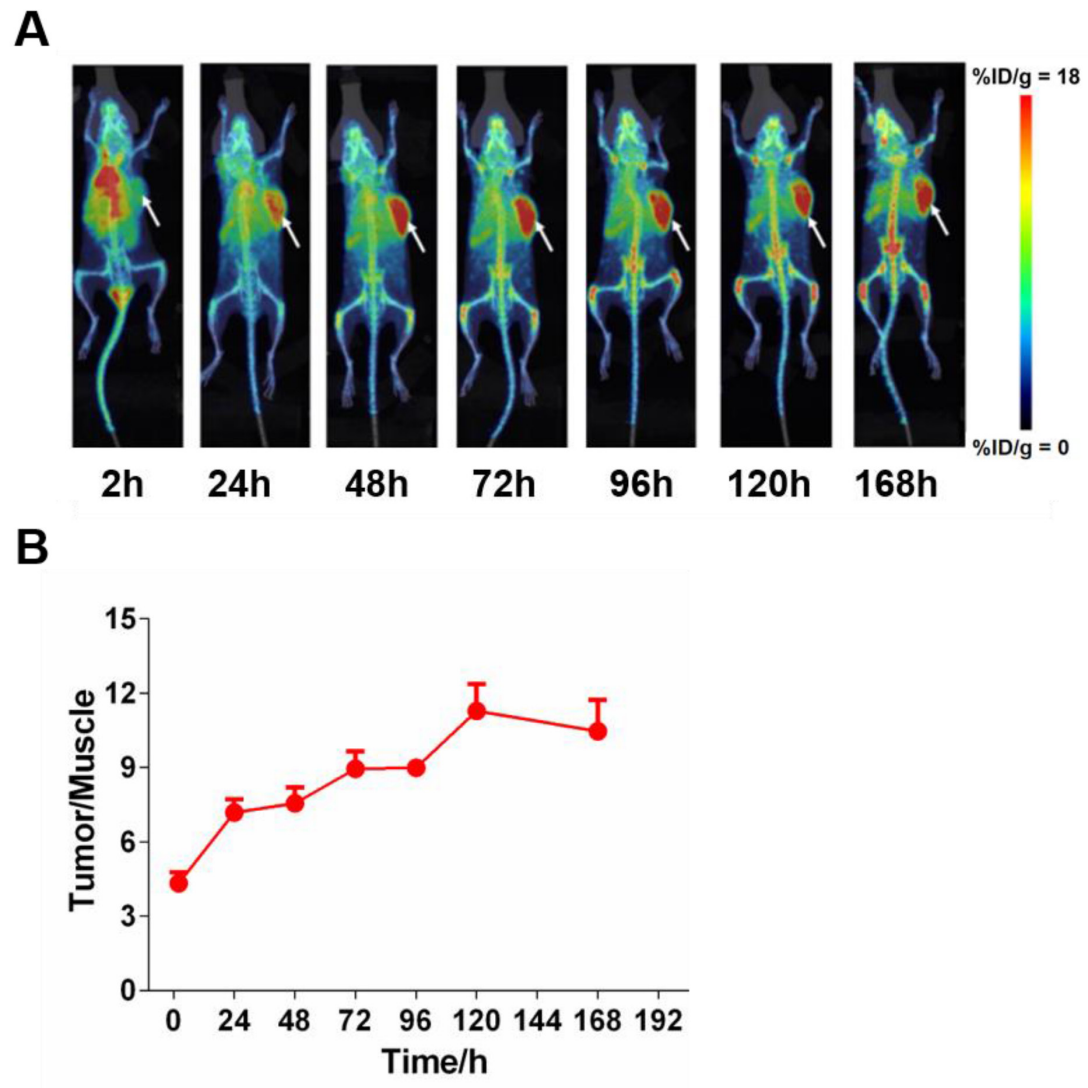
** Tissue distribution of 6MW3211 in MC38-hPD-L1/hCD47 tumor-bearing mice. (A)** Coronal maximum intensity projection images of tumor-bearing mice treated with single intravenous administration of ^89^Zr labeled 6MW3211 (200 μg/mouse) at different time points. **(B)** The tumor-to-muscle versus time curve in MC38-hPD-L1/hCD47 tumor-bearing mice after a single intravenous administration of ^89^Zr labeled 6MW3211 (n = 6). The average tumor-to-muscle ratio showed a trend of rising at first, followed by a decline, reaching its peak value of 11.28 at 120 h post-dose. The tumor/muscle values were showed as Mean ± SME.

To assess the tissue distribution characteristics of 6MW3211 *in vivo*, radioisotope (^89^Zr) labeled tracer method was used. After a single intravenous administration of ^89^Zr labeled 6MW3211 in B-hPD-L1/hCD47/hSIRPα triple transgenic mice inoculated with MC38-hPD-L1/hCD47 cells. PET/CT scanning was performed to detect isotope distribution. 6MW 3211 was mainly distributed in tumors and organs that are rich in blood flow (heart, liver, spleen, lung and kidney) and joints (Figure 5A). The distribution in other organs (brain, shinbone and muscle) was low (Figure 5A). Maximum radioactivity concentration was reached in tumors at approximately 72 h post-dose. A relative stable radioactivity level was maintained in the tumors (79.7% of the maximum value) at 168 h post-dose, while a decreasing trend was observed in most other organs examined. The average tumor-to-muscle ratio showed a trend of rising at first, followed by a decline, reaching its peak value of 11.28 at 120 h post-dose (Figure 5B). The low radioactivity in the brain indicates that ^89^Zr-labeled 6MW3211 cannot penetrate the blood-brain barrier. A gradual increase in radioactivity concentration was noted in joint, which may be related to the distribution of the unchelated ^89^Zr ion. These results indicated that 6MW3211 selectively bind to hPD-L1/hCD47 expressing tumors cells *in vivo*.

### Pharmacokinetic and safety profile of 6MW3211 in rhesus monkeys

**Figure 6:**
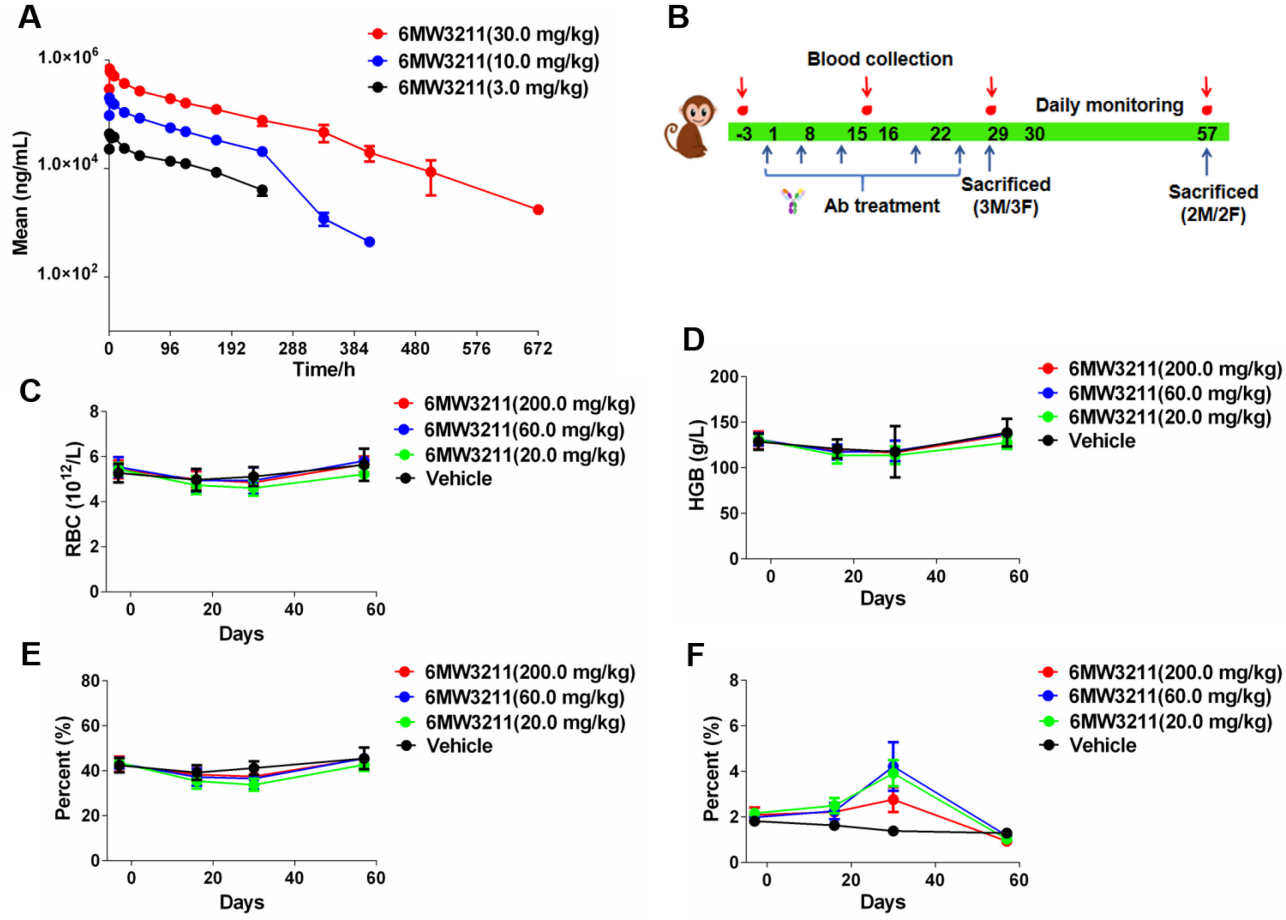
** Pharmacokinetic and toxicokinetic profile of 6MW3211 in rhesus monkeys. (A)** Mean PK profile of 6MW3211 in rhesus monkeys. A single dose of 6MW3211 (3, 10 and 30 mg/kg) were intravenously administered to rhesus monkeys, and the serum samples at different time points were collected and tested. **(B)** The schematic diagram of NHP toxicity study of 6MW3211. **(C)** The changes of RBCs in response to treatment with different doses of 6MW3211. **(D)** The changes of hemoglobin (HGB) in response to treatment with different doses of 6MW3211. **(E)** The changes of hematocrit (HCT) in response to treatment with different doses of 6MW3211. **(F)** The changes of reticulocyte (RET) in response to treatment with different doses of 6MW3211. All data values showed as Mean ± SEM.

The PK profile of 6MW3211 was characterized in rhesus monkeys. Following a single intravenous infusion of 6MW3211 at 3, 10 or 30 mg/kg, no consistent and obvious gender differences of systemic exposure were observed. Both C_max_ and AUC_0-240h_ increased more than dose-proportionally over the dose range of 3 to 30 mg/kg. The range of average elimination half-life (T_1/2_) across dose levels was 81.6 to 110 h, clearance (CL) was 0.513 to 0.806 mL/h/kg, volume of distribution (Vd) was 76.3 to 91.3 mL/kg, C_max_ was 45.447 to 690.556 µg/mL, and AUC_0-240h_ was 3.28 to 48.6 h*mg/mL in this study (Figure 6A). The volume of monkey plasma is assumed about 44.3 to 66.6 mL/kg, these data suggested that 6MW3211 was distributed beyond the circulation system.

The potential risk to humans receiving 6MW3211 was evaluated in rhesus monkeys in a GLP-compliant 4-week consecutive repeat-dose toxicity study (QW × 5) with a 4-week recovery period. Under the condition of this study, repeated intravenous injection of 6MW3211 were administrated in rhesus monkeys at doses of 20, 60 or 200 mg/kg once a week for 4 weeks (total 5 doses) (Figure 6B). All dose levels were clinically tolerated, with no notable clinical signs. No change was considered as adverse effects since the counts of RBC, levels of HCT and HGB in all monkeys differed within 20%, and the percentage of RET rose temporally but recovered in further period (Figure 6C-F).

No other clinical symptoms, such as hematological toxicities and evidence of target organ toxicity was observed for 6MW3211 treated animals, and the no-observed-adverse-effect-level (NOAEL) was determined to be 200 mg/kg. This NHP study indicated that 6MW3211 has very low risks of hematological toxicities compared to other CD47-based therapeutics.

### Co-expression of PD-L1 and CD47 on tumor tissues

**Figure 7:**
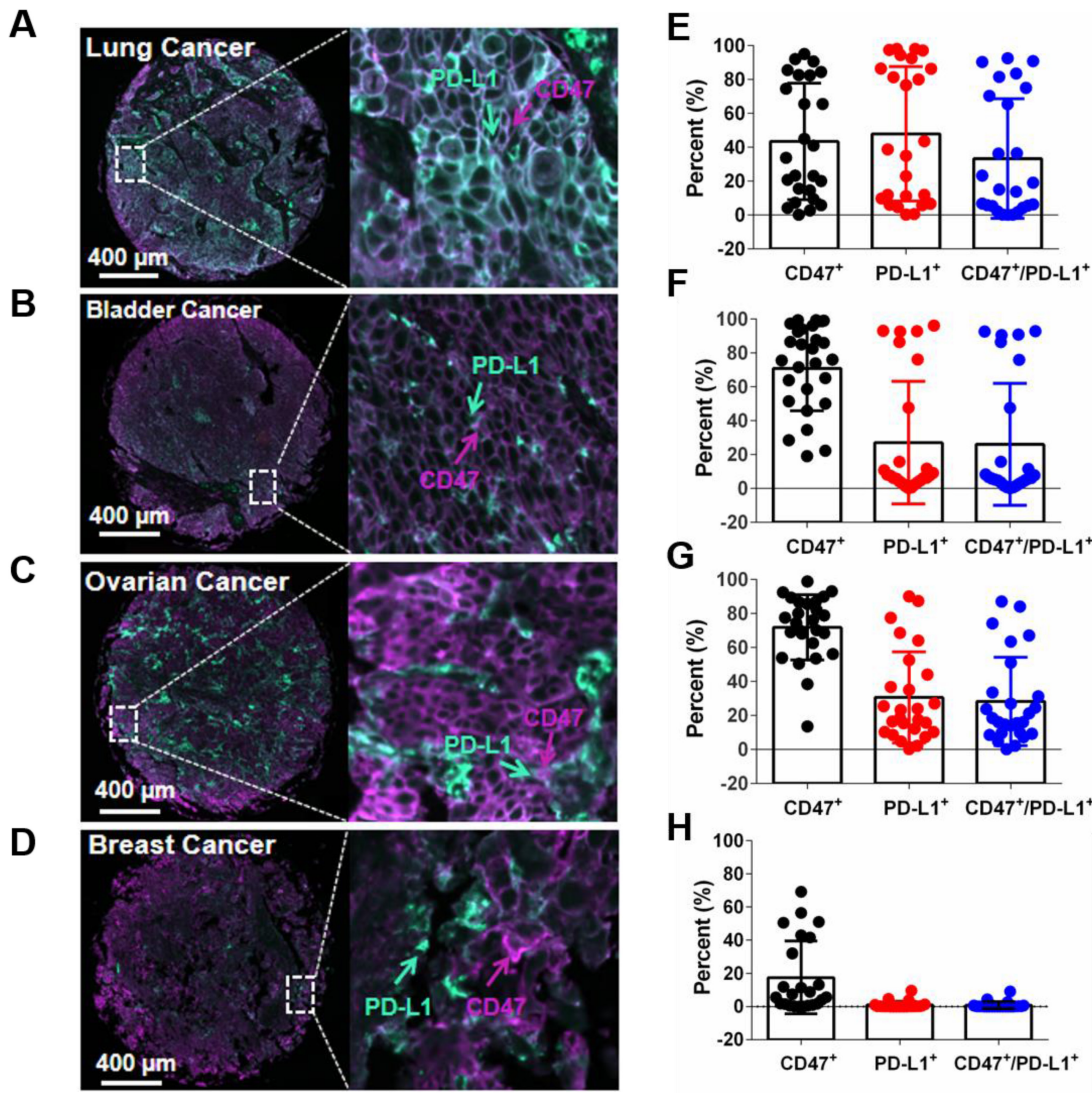
** Detection of the co-expression of CD47 and PD-L1 in different types of tumor tissues. (A-D)** Multiplex immunofluorescence images displaying the co-expression PD-L1 and CD47 in ovarian cancer, lung cancer, bladder cancer and breast cancer tissues. The scale bars represent 200 μm and 10 μm, respectively. **(E-H)** Expression of PD-L1 and CD47 in different types of tumor tissues. Data points, each representing one tumor tissue, are shown by solid circles.

To assess the prevalence of PD-L1 and CD47 co-expression on tumor cells, 96 human tumor tissues (24 tissues for each cancer type including breast cancer, ovarian cancer, lung cancer and bladder cancer) were evaluated by multiplex fluorescent immunohistochemistry (mIHC) staining on tumor tissue microarray (TMA) slides. A four-color fluorescent IHC kit was used and it enabled the simultaneous staining of three proteins and the nuclei on one TMA slide. Antibody against PanCK was applied to determine tumor cells. Co-expression of hPD-L1 and hCD47 was observed on several tissue samples on ovarian cancer, lung cancer and bladder cancer tissues, but not obviously on breast cancer tissues (Figure 7A-D).

Of the 24 lung cancer tissues, 11 showed more than 20% hPD-L1/hCD47 double positive tumor cells (11/24, 45.8%). For bladder cancer and ovarian cancer tissues, the percentages of more than 20% hPD-L1/hCD47 double positive tumor cells in one tissue are 29.2% (7/24) and 50% (12/24), respectively (Figure 7E-H). In contrast, very week or no staining of hPD-L1/hCD47 double positive tumor cells were found in human breast cancer tissues (Figure 7H). These data confirmed the co-expression of PD-L1 and CD47 on several tumor tissues, suggesting dual blockade of PD-1/PD-L1 and CD47/SIRPα signals by 6MW3211 would offer more clinical benefits than single PD-L1 or CD47 blockade.

## Discussion

By now, PD-1 is the most widely studied immune checkpoint inhibitory receptor, and more than 1000 clinical trials are ongoing to evaluate the efficacy of PD-1/PD-L1 blockades in a variety of cancers 6. Tumor cells with or without cytokine stimulation would express PD-L1 to inhibit T cell mediated cytotoxicity by engagement with PD-1 on T cell infiltrated into the tumor microenvironment 26. Then, PD-L1 expression became the criteria for patient's enrollment during all PD-1 based therapeutics.

As PD-1 expresses on T cell after its activation, PD-1/PD-L1 signaling was considered as the key signaling for adaptive immunity. In clinical trials, patients who experienced positive response are often have enough tumor specific T cell proliferation, activation, and tumor microenvironment infiltration 27, 28. For these patients with cold tumor or with enough inactivated T cell infiltration, boosting myeloid cell innate immunity to kill tumor cells followed by presentation of tumor specific antigen for T cell activation may enhance the anti-tumor activity for PD-1/PD-L1 blockades 29, 30.

CD47 is highly expresses on several hematological malignancies and solid tumors to inhibit innate immunity of myeloid cells 31-33. Both *in vitro* and *in vivo* studies showed that CD47/SIRPα axis is the key signal for macrophage phagocytosis and tumor antigen presentation 17, 34. As reported, CD47 not only expresses on macrophages, but also dendritic cells. Dr. Yang-Xin Fu's lab reported that blocking CD47 signaling in dendritic cells could decrease the degradation of double-strand DNA in cytosol, then activate cGAS-STING signaling to secret type-I interferons for dendritic cell maturation and migration 35. Except for tumor cells, RBCs also expresses high levels of CD47 to deliver negative signals to protect RBCs from macrophage phagocytosis. The ideal CD47-based therapeutic modalities could specifically activate macrophage phagocytosis of tumor cells but not RBCs followed by T cell activation. With the determination of CD47/SIRPα complex structure, two glycosylation sites were identified 36. Recently, several groups reported some SIRPα mutants and CD47 antibodies showed different binding ability to CD47 on tumor cells and normal cells, indicating different glycosylation model on CD47 in malignancy and normal cells. According to this theory, our team generated several tumor CD47 specific antibodies by using tumor cells and RBCs for FACS counter screening.

In this study, we generated a bispecific antibody 6MW3211, which blocks both PD-1/PD-L1 and CD47/SIRPα pathways to boost both innate and adaptive immunity for cancer patients. The anti-PD-L1 arm is high affinity to PD-L1 expressing on tumor cells, and the anti-CD47 arm is low affinity to reduce the potential cytotoxicity even in high concentration. Most importantly, the anti-CD47 arm of 6MW3211 selectively binds to CD47 on tumor cells but not CD47 expressing on RBCs, which may enable the good safety profile of 6MW3211 in NHP studies and further human clinical trials. Even though the anti-CD47 arm is low affinity, this arm could induce the macrophage phagocytosis of tumor cells both *in vitro* and *in vivo*. Once the anti-PD-L1 arm binds to tumor cells, the anti-CD47 arm can effectively disrupt the interaction of CD47/SIRPα and further inhibit tumor progression. Compared with other anti-CD47 or CD47/PD-L1 bispecific antibodies, 6MW3211 did not show any binding signal to human RBCs even in high concentration condition. In the human CD47 transgenic mouse and rhesus monkey models, at the dose of 20 mg/kg or 200 mg/kg condition, no red blood cell toxicity was observed. As for PD-L1 based therapeutics, high dose was needed to achieve positive clinical response. 6MW3211 was confirmed the specific binding to CD47 on tumor cells but no cross with CD47 on RBCs at high dose condition (>20.0 mg/kg). Notably, we demonstrated the co-expression of CD47 and PD-L1 on several tumor tissues using mIHC technology. This data is pretty informative for patients screening in clinical trials. For CD47 and PD-L1 double positive patients, 6MW3211 may preferentially bind to tumor cells followed by the activation of both innate and adaptive immunity. Targeting two different innate and adaptive checkpoints using a bispecific approach could synergistically remodel the tumor microenvironment that promotes potent anti-tumor activity. The therapeutic efficacy and safety profile of 6MW3211 in human patients will be further evaluated in clinical trials.

## Supplementary Material

Supplementary figures and tables.
