# Supplementary material for: Blockade of dual immune checkpoint inhibitory signals with a CD47/PD-L1 bispecific antibody for cancer treatment
Source: Theranostics. 2023 Jan 1;13(1):148–60. doi: 10.7150/thno.79367 (PMC9800731; doi:10.7150/thno.79367)
Supplement: Supplementary file 1 — Supplementary figures and tables. [file thnov13p0148s1.pdf]

Title:

Blockade of dual immune checkpoint inhibitory signals with a CD47/PD-L1 bispecific antibody for cancer treatment

Authors:

Rongjuan Wang[1, 2#], Chang Zhang[1, 2#], Yuting Cao[1#], Junchao Wang[3#], Shasha Jiao[1, 2], Jiao Zhang[1, 2], Min Wang[1], Peipei Tang[1], Zijun Ouyang[1], Wenlu Liang[1], Yu Mao[1], An Wang[1], Gang Li[1], Jinchao Zhang[1], Mingzhu Wang[3✉], Shuang Wang[1, 2✉], Xun Gui[1✉]

1. Mabwell (Shanghai) Bioscience Co., Ltd., Shanghai 201210, China.
2. Beijing Kohnoor Science & Technology Co., Ltd., Beijing 102206, China.
3. School of Life Sciences, Anhui University, Hefei 230022, China.

# These authors equally contributed to this article.

✉Corresponding Authors: **Mingzhu Wang,** E-mail: wangmzh@ahu.edu.cn, School of Life Sciences, Anhui University, Hefei, China. **Shuang Wang,** E-mail: ws@bjkohnoor.com, Beijing Kohnoor Science & Technology Co., Ltd., Beijing China. **Xun Gui,** E-mail: xun.gui@mabwell.com, Mabwell (Shanghai) Bioscience Co., Ltd., Shanghai, China.

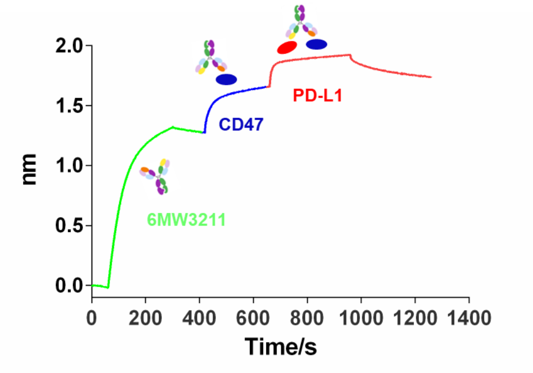

Figure S1. Simultaneously binding of 6MW3211 to CD47 and PD-L1 was measured by Octet RED96 system. 6MW3211 (4 μg/mL) was captured by AHC biosensors followed by flowing CD47 (60 nM) for 300 s and PD-L1 (200 nM) for 240 s.

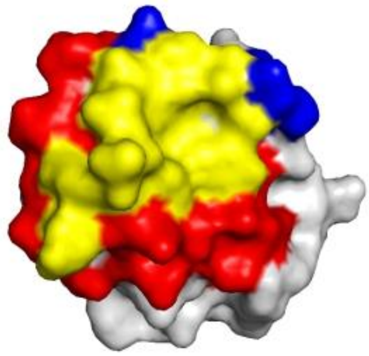

Figure S2. Regions of CD47 surfaces binding to SIRPα and 6MW3211-CD47 Fab. Red represents regions only binding to SIRPα, blue represents regions only binding to 6MW3211-CD47 Fab, and yellow represents regions binding to both SIRPα and 6MW3211-CD47 Fab.

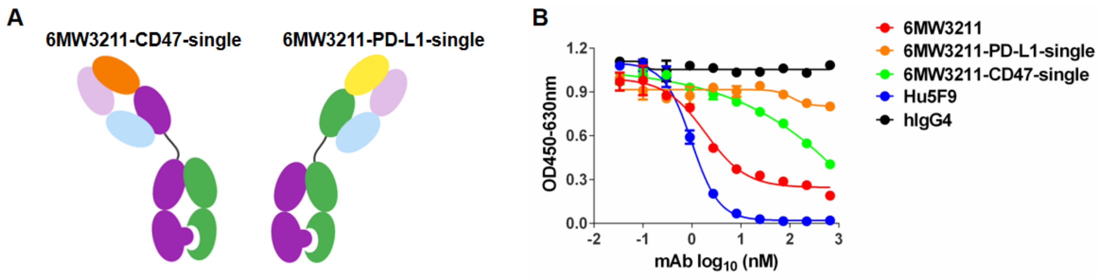

Figure S3. Blocking of CD47-SIRPa interaction by different antibodies. (A) Schematic diagram of 6MW3211-CD47-single and 6MW3211-PD-L1-single. **(B)** CD47-SIRPa blocking assay was performed by ELISA. PD-L1 recombinant protein was co-coated in the ELISA plates. Hu5F9, 6MW3211-CD47-single, 6MW3211-PD-L1-single and hIgG4 were used as control.

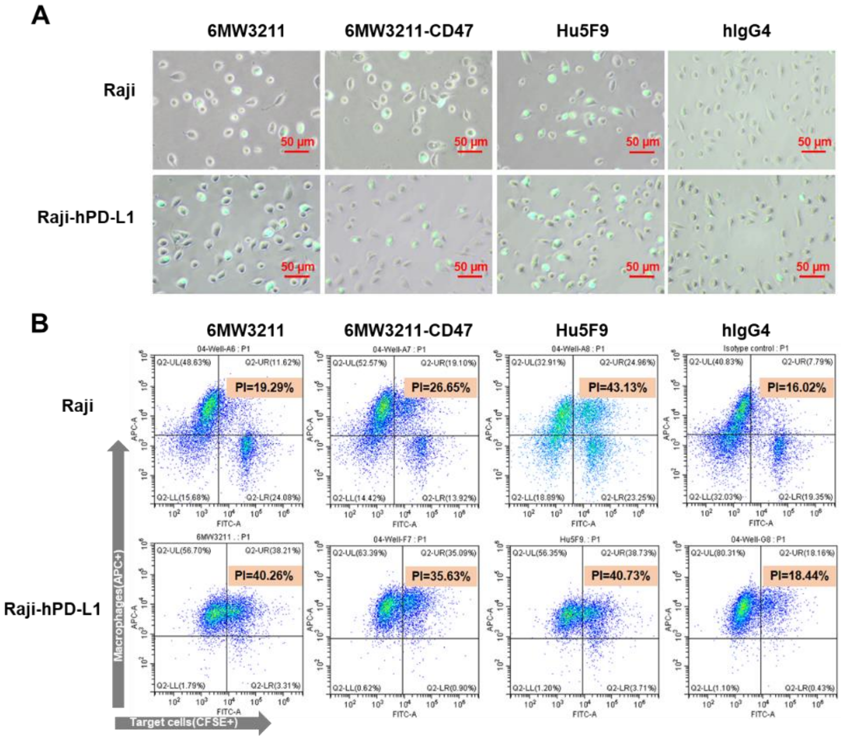

Figure S4. 6MW3211 promoted macrophage phagocytosis. The E/T ratio used for this assay was 1:5. 1.6 × 10^5 macrophages and 8.0 × 10^5 target cells were used for each test, and the antibody concentration used for this experiment was 132 nM. **(A)** The immunostaining images of macrophage phagocytosis. Target cells were stained with CFSE (Cat:423801, Biolegend) at 5 μM/well, incubated at 37 ℃ for 0.5 h. Immunofluorescence was observed after incubation with 6MW3211 for 1 h. **(B)** Flow cytometry diagram of phagocytosis assay. Cells were collected, added anti-MouseF4/80-APC antibody (Cat.E-AB-F0995E, Elabscience) with 5 μL/Test. After incubation for 0.5 h, the cells were centrifuged and resuspended in 200 μL PBS, and detected by flow cytometry. Phagocytic Index(PI)=Q1-UR/(Q1-UL+ Q1-UR)*100%.

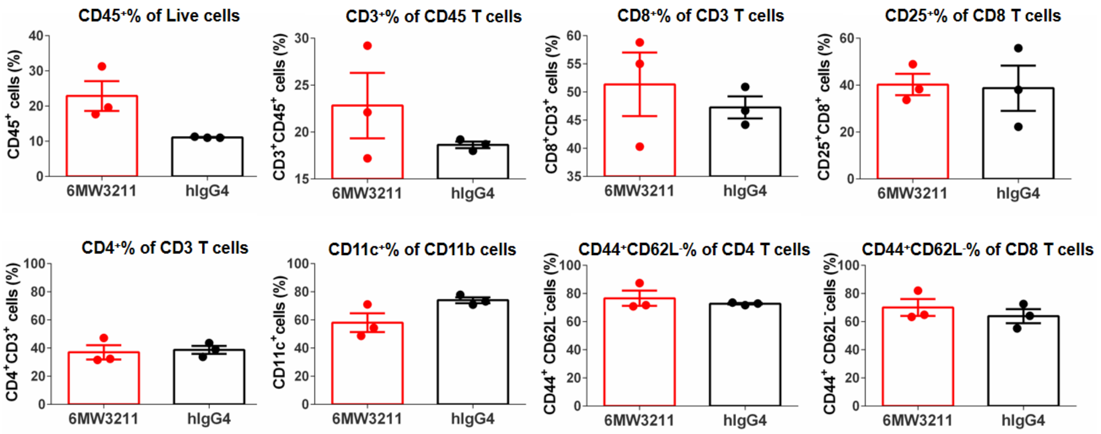

Figure S5. Immune cells infiltration in tumor tissues in MC38-hPD-L1/hCD47 (B-hPD-L1/hCD47/hSIRPα triple transgenic mouse model. 6MW3211 and hIgG4 treatment group (n = 3) were selected for tumor immune cells infiltration analysis after administration.

Table S1. Data collection and refinement statistics

	CD47-6MW3211 Fab
**Data collection**	
Wavelength (Å)	0.9785
Space group	$P2_12_12_1$
Cell dimensions	
a, b, c (Å)	60.83, 100.48, 163.32
α, β, γ (°)	90, 90, 90
Resolution (Å)	50.00–2.60 (2.69–2.60)*
R_{merge}	0.129 (1.010)
I/σI	17.6 (2.2)
$CC_{1/2}$	0.994 (0.828)
Completeness (%)	99.9 (100.0)
Redundancy	9.8 (10.4)
Total/Unique reflections	311,060/31,874
**Refinement**	
Resolution (Å)	40.57–2.60
R_{work}/R_{free}	0.187/0.231
No. of reflections used	31543
No. of atoms	
Protein	4380
Ligands	102
Water	252
Average B-factor (Å^2)	
Protein	35.0
Ligands	75.0
Water	34.3
R.m.s. deviations	
Bond lengths (Å)	0.004
Bond angles (°)	0.695
Ramachandran plot	
Favored (%)	96.9
Allowed (%)	3.1
Outliers (%)	0.0

Table S2. 6MW3211-CD47 Fab/hCD47 interactions

hCD47		Heavy chain		Light chain		Interaction
Region	Residue	Region	Residue	Region	Residue	
N-ter	Q1	CDR2	Y54			HB
BC loop	N32 (FUC)	CDR3	F102			VDW
				CDR2	Y49	VDW
				CDR2	S56	HB
	T34	CDR3	D101			HB
		CDR3	F102			HI
	E35	CDR3	D101			HB
		CDR3	F102			VDW
		CDR3	Y103			VDW
βC	V36	CDR3	Y103			VDW
	Y37	CDR3	Y103			HI
βC'	D51	CDR3	Y103			HB
C'C" loop	A53	CDR3	F102			HI
		CDR3	Y103			HI
		CDR3	L109			HI
	L54	CDR3	L109			HI
				CDR2	Y50	HI
				CDR2	R53	HB
βC"	K56			CDR1	Y32	HB (via water)
				CDR2	Y50	HB
βF	T99	CDR3	Y103			HI
FG loop	L101	CDR3	F102			VDW
		CDR3	Y103			VDW
		CDR3	A104			VDW
	T102	CDR1	T30			HB
		CDR1	N31			VDW
		CDR1	Y32			VDW
		CDR1	V33			VDW
		CDR2	N52			HB, VDW
βG	R103	CDR2	Y54			HI
